# Routes to colorectal cancer in Lynch syndrome: a decade of molecular and clinical insights converging on Schrödinger’s cat

**DOI:** 10.1007/s10689-026-00593-w

**Published:** 2026-08-04

**Authors:** Aysel Ahadova, Fatima Fayyaz, Leon Marin Grez, Hendrik Bläker, Matthias Kloor

**Affiliations:** 1 Department of Applied Tumor Biology, Institute of Pathology, Heidelberg University Hospital, Heidelberg, Germany; 2 Department of Precision Medicine, University of Campania ‘Luigi Vanvitelli’, Naples, Italy; 3 Institute of Pathology, University Hospital Leipzig, Leipzig, Germany

**Keywords:** Colorectal carcinogenesis, Cancer pathways, Cancer evolution, Adenoma, Lynch syndrome, Microsatellite instability

## Abstract

Colorectal cancer (CRC) development in Lynch syndrome (LS) has long been regarded to follow an adenoma-carcinoma sequence accelerated in comparison to microsatellite-stable (MSS) CRC development. Yet, several clinical observations challenged this hypothesis, most notably the persistently high CRC incidence under colonoscopy surveillance, a non-measurable benefit from shorter screening intervals, and substantial differences in cancer risk between carriers of the different mismatch repair (MMR) genes. Advances in the last decade have produced new hypotheses, highlighting the distinct biology of LS CRC and unravelling several co-existing pathways to cancer. Alongside the genomic heterogeneity, the immunogenic load created by the accumulation of frameshift mutations imposing selective pressure and leading to immune evasion, appears to be a critical step for cancer manifestation. We discuss potential clinical implications of these advances in understanding CRC pathogenesis for cancer prevention in LS and outline open questions that remain.

## Introduction

**Fig. 1:**
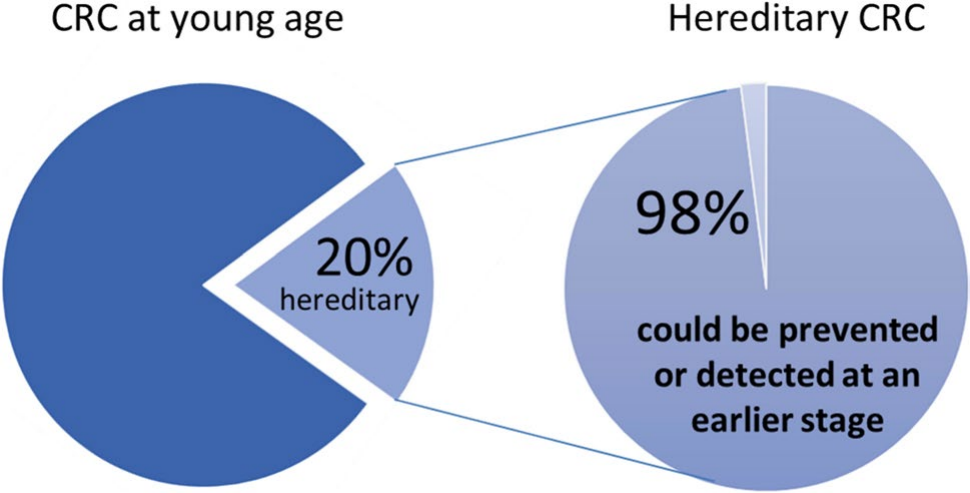
Proportion of hereditary colorectal cancer cases among patients under the age of 50 and the resulting potential to prevent tumors. One in five patients diagnosed with colorectal cancer under the age of 50 carries a tumor-associated germline variant [2]. Most of these tumors could be prevented or detected earlier if the hereditary predisposition were known and appropriate preventive measures were taken [9]

Hereditary predisposition is responsible for approximately 2–5% of all colorectal cancers (CRC) [1]. However, in younger people, the proportion of CRCs caused by a genetic predisposition is substantially higher: among CRC patients under the age of 50, every fifth has a germline genetic variant increasing their CRC risk (Fig. 1) [2]. The most common genetic cancer predisposition syndrome is Lynch syndrome (LS) affecting 25 million individuals worldwide [3–6]. Carriers have a significantly elevated risk of developing a broad spectrum of cancers, most commonly affecting the colorectum and endometrium [1, 7, 8]. Awareness of an existing genetic cancer predisposition allows timely interventions, which have the potential to prevent or intercept cancer development or to facilitate its early detection [9–11]. However, our understanding of the effective prevention is undergoing a massive transformation on the background of emerging evidence on molecular carcinogenesis of LS CRC.

LS is caused by a monoallelic pathogenic germline variant in one of the four MMR genes (*MLH1, MSH2*, *MSH6*, or *PMS2*) [12]. In addition, large terminal deletions in the epithelial cell adhesion molecule gene *EPCAM* cause transcriptional readthrough and subsequent epigenetic silencing of its structurally intact neighbour *MSH2*, observed in 2–3% of LS patients [12–14]. Studies also reported a heritable epigenetic abnormality termed constitutional *MLH1* epimutation, which involves monoallelic hypermethylation of the *MLH1* promoter and the associated allele-specific loss of expression throughout somatic tissue, to cause LS (less than 1% of cases) [15].

### Epidemiology of CRC in LS carriers: before surveillance and after surveillance

Large prospective cohort studies have improved our understanding of CRC epidemiology in LS in the era of regular surveillance and have produced reliable gene-specific estimates of CRC risk comprehensively summarized in a recent meta-analysis [5, 6]. Strikingly, colonoscopy surveillance, implemented in LS carriers (previously ascertained based on family history alone and called hereditary non-polyposis colorectal cancer, HNPCC) as a preventive measure without prior effectiveness testing in randomized clinical trials, has revealed previously unknown features of LS carcinogenesis explaining the distinct clinical presentation of LS CRC [16].

Colonoscopy remains the gold standard approach for CRC interception in LS carriers and a major contributor to the reduced CRC-related mortality [17–20]. However, in contrast to the expectation of CRC prevention, multiple independent studies reported surprisingly high CRC incidence despite colonoscopy [20–23]. This observation culminated in the recent comparison study between the International Mismatch Repair Consortium (IMRC) and the Prospective LS Database (PLSD) data. Comparing a prospective cohort of patients who underwent colonoscopy with a retrospective cohort of patients, most of which did not have a baseline colonoscopy, the study showed similar CRC incidence [8, 24, 25]. This further fuelled the debate on the possible reasons of a potentially limited CRC-preventing effect of colonoscopy in the LS setting, which have been previously discussed in Ahadova et al. [26].

The prerequisite for colonoscopy to perform well as a tool for CRC prevention is the presence of endoscopically detectable precancerous stages. Ideally, these precancerous stages should have a dwell time compatible with reasonable intervals between colonoscopy exams. The understanding of the precancerous stages in LS was initially shaped by the Vogelstein model of CRC development drawn from the specific example of Familial Adenomatous Polyposis (FAP), and extrapolated to all CRCs [27]. This model assumed a gradual progression from normal cells acquiring Wnt activating mutations, typically in the *APC* gene, and considered MMR deficiency to be an “accelerator” of the mutational processes after the acquisition of canonical CRC drivers. Most importantly, this model assumed a trajectory of CRC development going through a precancerous stage characterized by visible polypous lesions. In the general population, CRC development is presumably taking decades [27], justifying CRC screening starting from the age of 50 (or 45, accounting for the recent trend towards early-onset CRC) and repeating every 10 years as a standard protocol commonly followed internationally [28, 29].

In patients with a genetic cancer risk, such as LS, the potentially malignant transformation kicks off earlier due to an inherited germline variant and is accelerated due to the increased mutation rate, however the exact dwell time of detectable precancers is unknown. This led to different surveillance strategies applied internationally varying from stricter regimens of yearly colonoscopy to more relaxed regimens of three-year intervals. The comparison of the various surveillance protocols surprisingly did not reveal any advantage of shorter colonoscopy intervals: neither CRC incidence nor tumor stage were lower in patients undergoing more frequent colonoscopies [30, 31]. This counter-intuitive observation challenged the accepted model of carcinogenesis in LS and highlighted the importance of better understanding the molecular routes of carcinogenesis.

### Who’s on first: the initiators of LS CRC

The possibility of MMR deficiency being the initiating event in LS carcinogenesis was questioned due to 2 observations: (1) the existence of adenomas with proficient MMR status in LS carriers [32–36], suggesting a later onset of MMR deficiency; (2) the absence of a strongly elevated adenoma risk in LS carriers, suggesting the contribution of MMR deficiency to the progression from adenoma rather than the initiation of the adenoma development [12, 37], again implying MMR deficiency occurring later. However, what seems to be a contradiction can easily be resolved by recognizing the existence of several independent routes to cancer. Consequently, this challenges the notion that adenoma formation represents the sole rate-limiting step in LS carcinogenesis. The discovery of MMR-deficient crypt foci (MMR-DCF) suggested the existence of a LS-specific precursor of a completely different nature: these putative “molecular” precursors do not show morphological or architectural alterations rendering them unsuitable for detection by colonoscopy, however they display the main molecular feature of almost any LS cancer, microsatellite instability (MSI) arising due to MMR deficiency [38–40].

Studying carcinogenesis and understanding the potential for malignant transformation is challenging as there are no means to longitudinally track the development of various lesions in the human scenario and to deduce the features determining their fate over time. However, it is possible to determine mutational signatures in manifest cancers and by that reconstruct the likely order of events during carcinogenesis [41]. Specifically, the impact of MMR deficiency as a mutational process could be quantified by analyzing the compatibility of MMR deficiency mutational signatures in mutational events strongly linked to initial steps of colorectal carcinogenesis. According to the Vogelstein adenoma-carcinoma model, mutations affecting the genes *APC*- > *KRAS*- > *TP53* constitute the classical sequence leading to cancer [27]. Using this sequence as a reference, mutational signatures of MMR deficiency were analyzed in a variety of somatic mutations in these genes. This transformed the classical view of LS carcinogenesis, because not only *KRAS* mutations (a predominance of G13D mutations followed by G12D, which is different from mutations typically observed in MSS CRC) but also the majority of *APC* mutations were compatible with mutational signatures of MMR deficiency, implying that MMR deficiency precedes *APC* mutation in a substantial proportion of LS CRCs [42]. Importantly, these data suggested that LS carcinogenesis cannot be reliably described by a single pathway: at least three pathways may lead to LS CRC, two of which are initiated in MMR-deficient lesions and the third one with MMR inactivation after adenoma formation. This view reconciles seemingly conflicting observations on the timely order of events and allows appreciating the heterogeneity of progression pathways [43, 44].

**Fig. 2:**
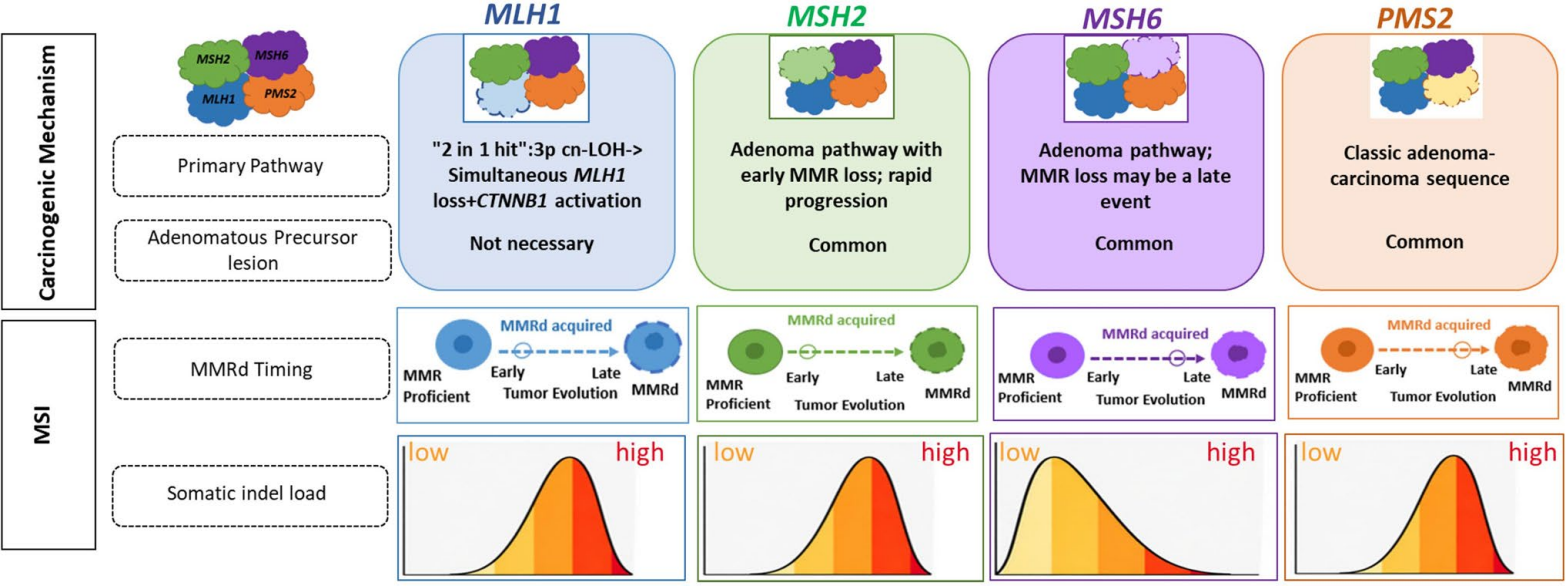
Heterogeneity of LS CRC development by MMR gene. Depending on the MMR gene affected in the germline, LS CRC development may differ in the primary carcinogenic pathway, presence of adenomatous precursor lesions prior to cancer, the timing of the MMR deficiency during the tumor progression and the somatic indel load

The “somatic heterogeneity” of pathways is consistent with the “germline heterogeneity” of LS caused by variants in MMR genes each having distinct roles and distinct degrees of essentiality/redundancy in DNA mismatch repair (Fig. 2). Here, particularly CRCs associated with the two low-penetrance genes, *MSH6* and *PMS2,* develop mostly through pathways characterized by late onset of MMR inactivation [45]. The comparison of *MSH6*- and *PMS2*-associated CRC revealed higher levels of single nucleotide variant (SNVs) and lower levels of insertion-deletions (INDELs) in *MSH6* vs *PMS2* CRCs, which might be linked to a less prominent role of MSH6 in INDEL repair due to partial functional redundancy with MSH3 resulting in lower degrees and later onset of MSI in *MSH6* LS CRC [46]. This was confirmed by follow-up studies showing lower coding microsatellite (cMS) mutation frequencies and mutant allele ratios [47] and shorter length shifts [48] in *MSH6* CRC compared to other LS CRC. Interestingly, in *PMS2* carriers, MSI manifests eventually in the majority of CRCs, however does not seem to be driving the initial steps of carcinogenesis. Thus, *PMS2* CRCs present with a similar cMS mutation profile and mutations frequencies [49], but do not seem to carry MMR deficiency mutational signatures in the canonical CRC drivers [50], and typically present with MMR-proficient adenomas [51, 52]. These observations align with the recent findings from the *MSH6* and *PMS2* carriers from Iceland showing real-world data from the pre-surveillance era and demonstrating absence of metachronous CRC in these carriers [53].

### Gene alliances: the relevance of genomic neighborhood and genetic context

Importantly, the intermediate stage of endoscopically visible polypous precursor, at least those with a clinically meaningful dwell time, does not seem to be a prerequisite for CRC development in LS. A subgroup of tumors initiated by MMR deficiency do not present with somatic *APC* mutations but show somatic *CTNNB1* mutations instead. Both acting in the Wnt signaling pathway, these mutations are typically mutually exclusive. However, whereas *APC* mutations are known to cause polyp formation, mutations in *CTNNB1* encoding for beta-catenin were associated with LS CRCs mostly lacking the evidence of polypous growth [54, 55]. Data on the proportion of CRCs with *APC* or *CTNNB1* mutations in each MMR gene group are scarce, and further studies of Wnt activating mutations in LS are warranted.

**Fig. 3:**
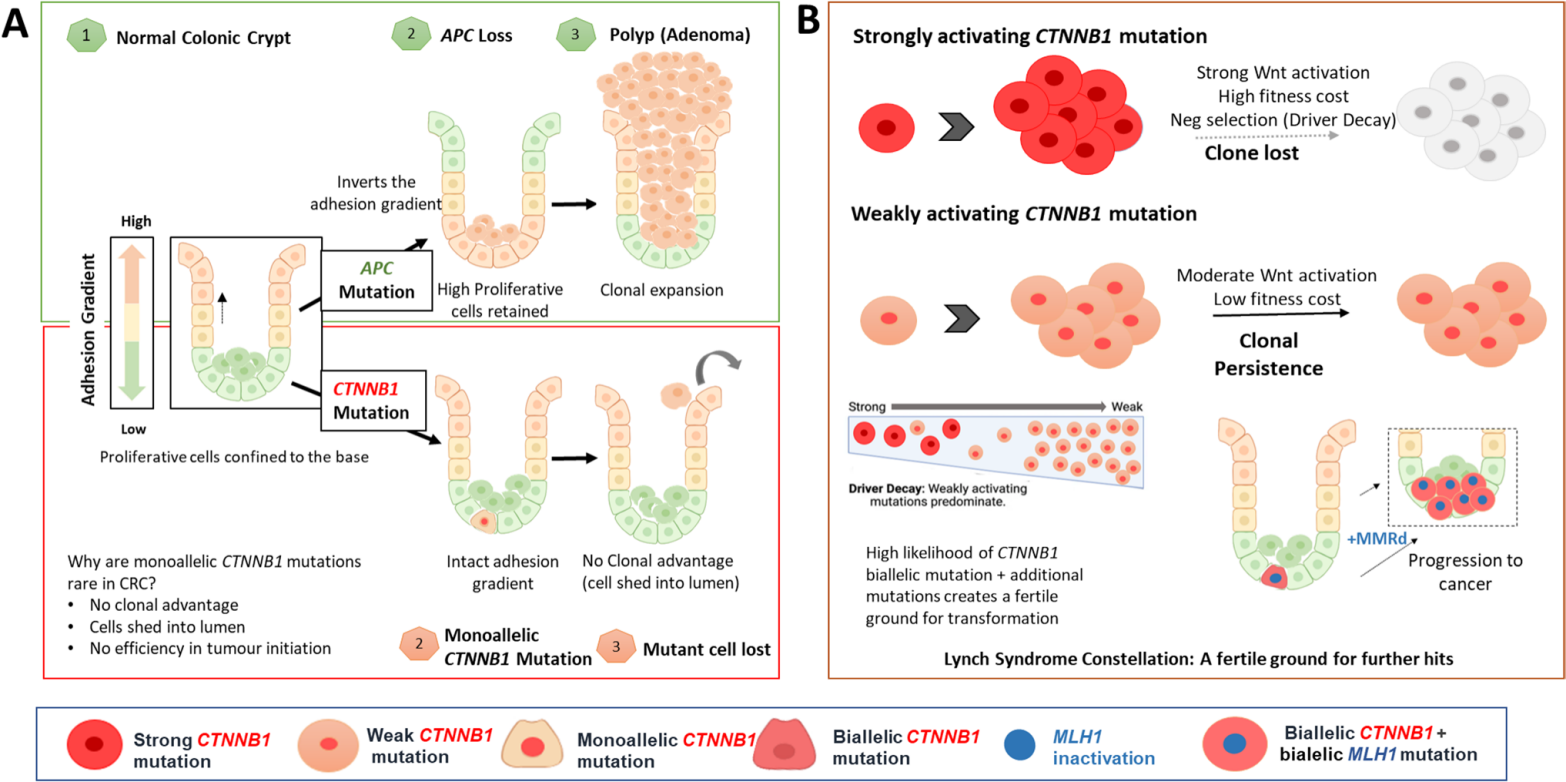
Impact of different *CTNNB1* mutations (monoallelic, biallelic, strongly and weakly activating mutations) on LS CRC development. **A**. In normal colonic crypts, proliferative cells remain at the crypt base, while differentiated cells migrate upward and are shed. APC maintains this spatial organization. APC loss disrupts this architecture, allowing proliferative cells to remain within the crypt and expand clonally. In contrast, monoallelic *CTNNB1* mutations activate WNT signaling but generally preserve normal cell migration and shedding, preventing stable crypt colonization and limiting clonal expansion. **B**. Driver decay hypothesis. Strongly activating *CTNNB1* mutations induce excessive WNT signaling and high fitness costs, resulting in negative selection and clonal extinction. Weakly activating mutations produce moderate WNT activation with lower fitness costs, enabling long-term persistence. In mismatch repair-deficient (MMRd) LS tissues, such persistent clones may acquire additional alterations, including biallelic *CTNNB1* and *MLH1* mutations, thereby promoting malignant progression. Thus, weak *CTNNB1* mutations may act as fertile early lesions that remain evolutionarily tolerated, particularly in MMRd tissues, thereby increasing the opportunity for secondary hits and cancer development

The recent findings from animal studies and mathematical modelling of *APC* and *CTNNB1* mutations have shed light on the potential reasons for the pattern described above, showing that these mutations though acting in the same pathway have different effects on the crypt architecture [56]. In the normal crypt, an increasing APC-dependent adhesion gradient toward the lumen ensures that cells with higher proliferative capacity experience differential adhesion forces that accelerate their upward migration and eventual extrusion. Whereas an *APC* mutation inverts the adhesion gradient along the crypt, causing the cells with higher proliferative capacity to remain in the crypt and thereby inducing polyp formation, *CTNNB1* mutations do not affect the adhesion gradient and are usually shed into the lumen. Consequently, *CTNNB1*-mutant cells, despite their increased proliferative signaling, are still efficiently displaced and eliminated (Fig. 3A) [56].

This could also provide a potential explanation to why monoallelic *CTNNB1* mutations alone are not capable of transforming the crypt, require additional hits and are extremely rarely found in CRC outside the LS context. In contrast, *APC* loss uncouples proliferation from directional migration by reversing the adhesion gradient, allowing mutant cells to persist within the crypt and initiate adenoma formation. The integration of this knowledge into the existing mathematical models and simulations of CRC development in LS scenario and beyond would provide a substantial added value [57–60]. Importantly, in LS a unique genomic constellation is created by the increased likelihood of the biallelic *CTNNB1* mutations accompanied by additional mutational processes explained below.

The initial hypothesis of CRCs developing from MMR-deficient crypts acquiring *CTNNB1* mutations has undergone certain transformation upon the discovery of unequal distribution of *CTNNB1*-mutant CRC in LS depending on the affected MMR gene, once again underlining the heterogeneity of LS carcinogenesis: CRC with *CTNNB1* mutations were predominantly observed among *MLH1*-associated CRC, presenting with somatic *CTNNB1* mutations in about half of cases, whereas *MSH2*-, *MSH6*- and *PMS2*-associated CRC showed a very low frequency of or no *CTNNB1* mutations at all [42, 46, 50, 61]. Importantly, *CTNNB1* mutations in *MLH1* CRCs are mostly biallelic, as monoallelic *CTNNB1* hits seem to be insufficient to transform colonic epithelium, according to the concept described above [56, 62, 63]. Interestingly, this molecular association of *MLH1* germline and *CTNNB1* somatic mutations echoed with the clinical observation of the high risk for incident CRC development despite low risk of adenoma in *MLH1* carriers, further fuelling the idea of potential CRC development skipping a polypous precursor stage with a sufficient dwell time for colonoscopy detection [61].

The reason for the close association between *MLH1* and *CTNNB1* is the genomic localization of these two genes on the short arm of the chromosome 3. Statistically seen, a biallelic oncogenic mutation is a very unlikely event as it would require a specific gain-of-function mutation to happen twice in the same cell. However, the genomic neighbourhood of *CTNNB1* with *MLH1* offers a favorable context resulting in a shortcut to malignant transformation: the close localization of the two genes enables a common event of copy number-neutral loss of heterozygosity (cnLOH) which duplicates not only the germline *MLH1* alteration but simultaneously also the first somatic mutation in *CTNNB1* [64, 65]. This requires the occurrence of an at least monoallelic *CTNNB1* mutation on the same chromosome as the germline hit in *MLH1* before the occurrence of the (cnLOH) event. The existence of monoallelic *CTNNB1* mutations in non-transformed colonic crypts has in fact been demonstrated previously [64], underscoring the plausibility of this hypothesis.

In the context of LS, where germline MMR mutations create tissue-wide susceptibility to an MSI phenotype upon a “second hit” inactivation of the remaining functional MMR allele, the pre-existing clonal architecture of the normal mucosa becomes critically important. In particular, the interaction between *CTNNB1* mutations and MLH1 loss in LS patients may represent one of the most striking pieces of evidence supporting the hypothesis of pre-existing driver fields in an otherwise phenotypically normal mucosa serving as a substrate for carcinogenesis. Notably, *CTNNB1* alterations enriched in *MLH1*-associated LS CRC mainly consist of S45 and T41-mutations, which are only weak to moderate activators of the WNT pathway [64, 66]. This preferential enrichment of weakly activating *CTNNB1* mutations could occur due to counter-selection of an overactive WNT-pathway post-cnLOH (the “just right” hypothesis) [67]. Alternatively, it may also be explained by the concept of driver mutation decay, according to which the weaker-activating mutations may have a longer dwell time compared to stronger-activating ones, enhancing their chance to undergo transformation (Fig. 3B)[68]. This highlights that the role of WNT-activation is highly contextual, as strongly activating *CTNNB1* mutations are likely counter-selected in normal mucosa, while the same mutations can be advantageous and selected when additional tumor drivers are present.

The integration of this concept results in the following putative sequence of events which yet needs to be validated by further research: *CTNNB1* mutations are initial somatic events, and either single crypts or fields harboring these mutations may serve as substrate for subsequent MMRd events. The type of *CTNNB1* mutations observed in manifest CRCs could be determined either by its “just right” WNT-activation or, alternatively, by the likelihood of that particular mutation to persist long enough to serve as a substrate for MMRd, or both. If these two are conceptualized as independent stochastic processes, then it appears far more likely for an *MLH1* LS CRC to arise on top of a weakly activating *CTNNB1* mutation rather than a strong one, simply because the latter is counter-selected in normal tissue so that its short persistence renders it far less likely as a substrate compared to a weakly activating mutation.

Notably, MMRd alone may not be sufficient to achieve fixation of the *CTNNB1* mutation. Instead, it is possible that the hypermutator phenotype is responsible for the induction of secondary driver mutations, which contribute to the persistence of *CTNNB1* mutations. In the MMRd scenario, commonly occurring inactivation of SMAD4 signaling, which has been shown to enable persistence of *CTNNB1* mutations, could play the role of such secondary drivers, as these mutations were found in all *MLH1* CRC with biallelic *CTNNB1* mutations [64, 69]. The relative contribution of each of the described possibilities remains an open question and needs to be addressed in the future.

### Lessons learned from molecular carcinogenesis

Understanding the molecular events determining the fate of developing tumor clones may also shed light on the factors influencing cancer risk in LS. LS carriers on average have a 50% lifetime risk of developing CRC, meaning that currently the scientific basis for predicting who will go on to develop a cancer and who will remain cancer-free is very thin. However, the potential determinants of cancer risk are emerging. One aspect is the likelihood of acquiring the second hit conferring MMR deficiency. Although studies have shown that this step is most likely the rate-limiting step during the malignant transformation, the factors influencing the chance of acquiring second hits are not fully understood [70, 71]. One factor is patient age, which increases the risk of chromosomal aberrations leading to the loss of the functional MMR gene allele, consistent with the epidemiological observation of increasing cancer risk at older age. Another so far unexplored possibility is the influence of environmental (dietary, epigenetic, drug-related etc.) factors capable of triggering chromosomal aberrations [71, 72]. These possibilities need to be addressed in future research.

Whereas tumors with chromosomal instability (CIN) traverse a karyotypic bottleneck and stabilize via negative selection [73], LS tumors may undergo an analogous bottleneck at the level of immunogenic mutation load [74–76]. The high mutational and neoantigen load of cells with MSI exerts great selective pressure on the emerging cancer. Under these circumstances, cancer can only manifest if the immune response against cancer is circumvented. One common mechanism of immune escape is the activation of checkpoint molecules on tumor-infiltrating lymphocytes, which leads to the exhaustion of the immune cells and disarms the host’s defense [77, 78]. The reversal of this mechanism by checkpoint blockade potentially resulting in complete regression of cancer across clinical stages shows the crucial relevance of this mechanism for the establishment of tumors in the LS context. In addition to reprogramming the immune environment, MSI cells may undergo reprogramming of their own, mostly affecting their capability to present antigens. Disruption of the antigen presentation is observed in 70% of all MSI cancers and restricts the immune response generating a “safe space” for tumor growth despite an intact immune microenvironment [79].

The antigen presentation can also undergo editing by the immune response eliminating highly immunogenic cell clones and leaving the space to the low immunogenic clones to grow out. Such selection of antigens to be presented may result from a long selection process on the background of the inherited HLA genotype. Analogous to the establishment of a ‘core karyotype’ in CIN tumors, LS-associated neoplasms may be defined early by a ‘core immunogenomic state’, a selected combination of mutations and immune-evasion features that together achieve a local fitness optimum and subsequently constrain further evolutionary trajectories.

It has been shown that the frequency of frameshift mutations in a tumor negatively correlate with the immunogenicity of the respective frameshift neoantigens derived thereof [75]. In fact, the initial phases of carcinogenesis may be associated with higher diversity of neoantigens with varying immunogenicty, which might then undergo immune elimination of the most immunogenic ones leading to the survival of only clones providing sufficient growth advantage without inducing immune elimination. Interestingly, there are some exceptions: mutations providing growth advantage crucial for the carcinogenic process and significantly outweighing the speed of immune elimination can survive without compromising on immune visibility [47, 75]. Importantly, this correlation seems to depend on the antigen presenting competence of the tumor cells: cells not carrying the necessary HLA molecules for presenting the respective antigens are less affected by this inverse correlation [47, 75, 80]. The antigen presenting competence can be linked to the acquired loss of crucial antigen presentation proteins, such as *B2M* mutations [79, 81], or constitutional constellation preventing certain antigens from being presented due to the lack of HLA molecules with suitable epitope-binding grooves [82, 83]. Thus, some LS carriers might have a less immune-aware constitution from birth on, which could modify their cancer risk in later life.

Applied to the epidemiologic observations, the immune elimination of arising cell clones might be the factor previously not accounted for but currently gaining more impact. If even clinically manifest cancers can disappear completely without any surgical intervention upon unleashing of the immune cells, it is conceivable that also preclinical cancers can undergo immune elimination if given sufficient time [84]. This hypothesis would explain why the CRC incidence does not decrease with shorter colonoscopy intervals [31, 85, 86]. It also shows how detection bias might influence our perception of cancer development and distort cancer risk estimations. Due to ethical considerations, it is not possible to design and perform a randomized controlled trial assessing the true impact of modern era colonoscopy and the surveillance intervals on cancer incidence in LS. However, considering the immune biology of MSI and applying Ockham’s razor, the available epidemiological evidence suggests that spontaneous clearance of precancerous lesions resulting in overdiagnosis at shorter colonoscopy intervals is the most likely explanation.

In analogy to observer effects in quantum physics, colonoscopy examination influences the observable state of disease by identifying lesions that would otherwise remain latent. In this sense, the situation resembles Schrödinger’s cat: until the “box” is opened by colonoscopy, a lesion may conceptually exist in multiple potential states, including progression, persistence, or spontaneous regression, yet observation collapses these possibilities into a single observable reality. The unobserved natural history of the lesions is therefore inherently inaccessible to direct study. In other words, colonoscopy does not merely observe lesions but defines which lesions become clinically observable. The impact of constant removal of the lesions which could train the immune system to recognize the neoantigens needs to be a topic for future research [87].

## Conclusion

LS offers a distinctive real-world framework for studying how tumors arise in a strongly immunogenic setting. The unique genomic constellations that permit otherwise highly unlikely mutational events give rise to non-canonical carcinogenic pathways and result in previously unknown clinical manifestations. These manifestations challenge the conventional understanding of colorectal carcinogenesis and are difficult to capture using methods developed for completely different biological contexts. However, elucidating the molecular basis of tumor development does not only advance the field of LS but also encourages the scientific community to reconsider long-held dogmas and adopt a fresh, less biased perspective on tumor evolution. Importantly, as previously predicted by Prof. Jeremy Jass [88], acknowledging the existence of multiple parallel pathways of cancer progression is mandatory to informing cancer prevention strategies in LS and beyond.

## Data Availability

No datasets were generated or analysed during the current study.
